# Supplementary material for: Genomic Analysis of Two Novel Bacteriophages Infecting Acinetobacter beijerinckii and halotolerans Species
Source: Viruses. 2023 Feb 28;15(3):643. doi: 10.3390/v15030643 (PMC10057805; doi:10.3390/v15030643)
Supplement: Supplementary file 1 [file viruses-15-00643-s001.zip › viruses-2187816-supplementary.pdf]

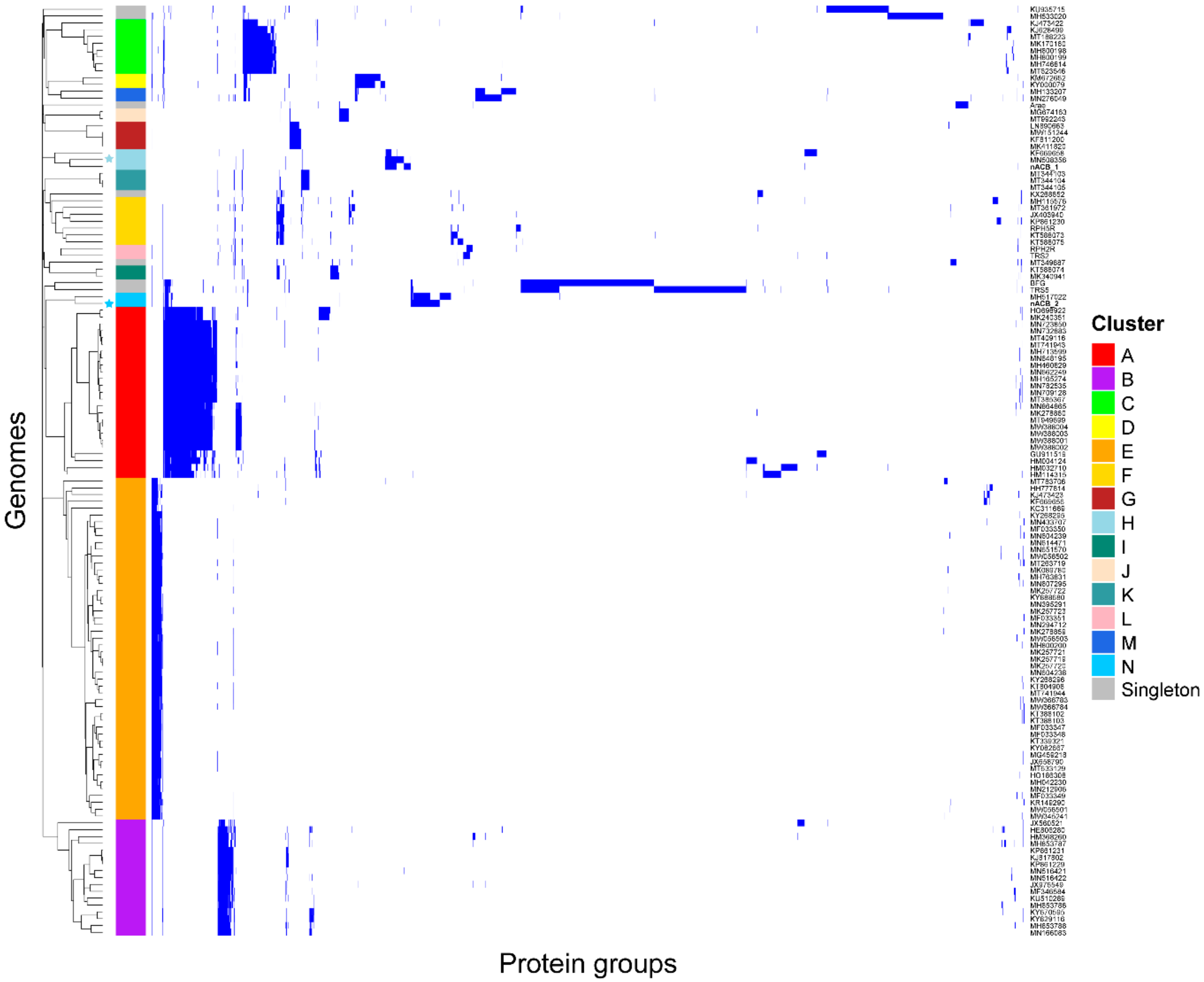

Figure S1. Heatmap illustrating the presence and absence of protein groups per phage genome. Presence or absence of each protein group is denoted in the heatmap by cells of blue and white colour, respectively. Protein groups were clustered using the UPGMA method of hclust in R. Phage genomes were hierarchically clustered using complete linkage. Coloured bars adjacent to the dendrogram represent clusters described in [6]. Phages nACB1 and nACB2 are indicated by the presence of a star adjacent to the tip label.

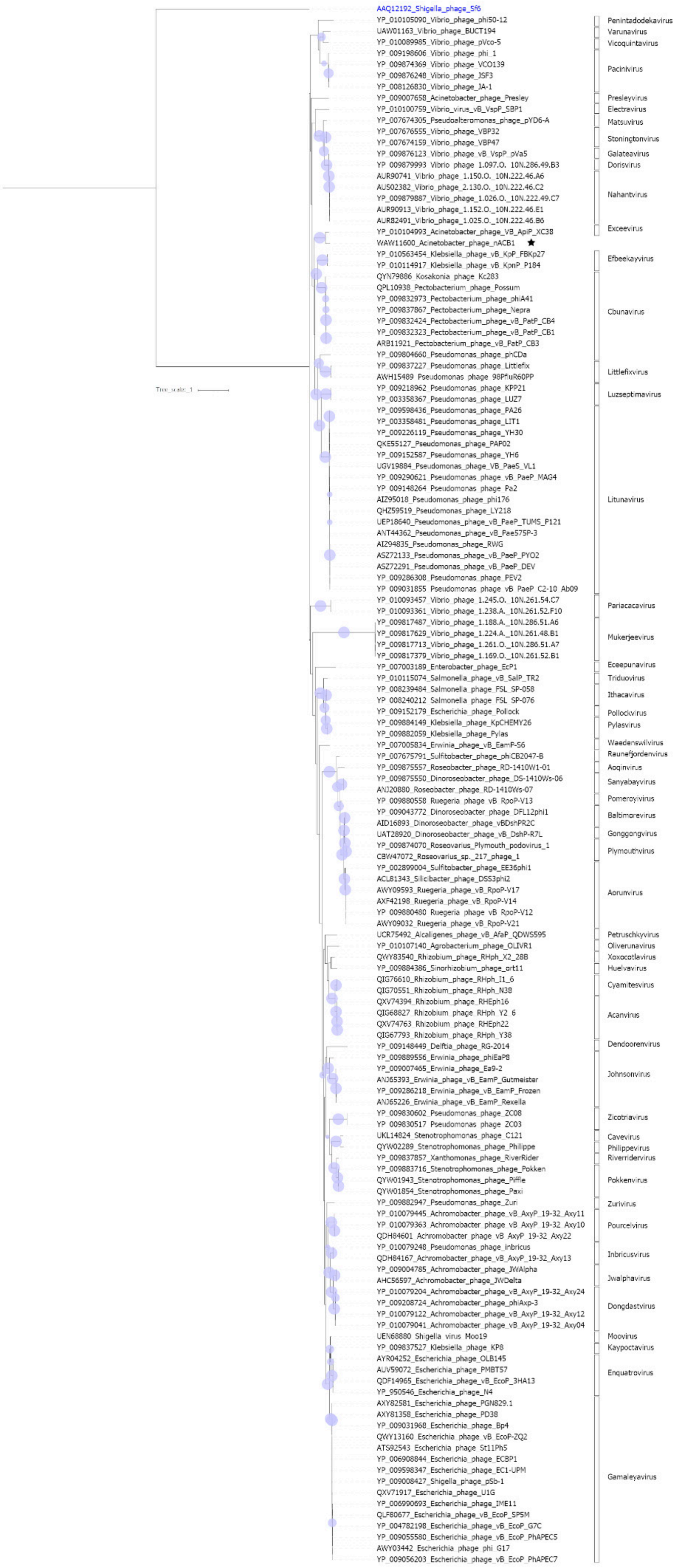

Figure S2. ***Schitoviridae* phylogenetic analysis of large terminase subunit proteins.** A log-likelihood of consensus tree - 34174.875 is presented with a substitution model LG+R5. Ultra-fast bootstrap >= 95% are shown as filled circles with the size proportional to the value. Bars adjacent to the labels indicate current International Committee on Taxonomy of Viruses (ICTV) genera. nACB1 is highlighted in bold font and star symbol. Outgroup sequences used to root the trees are coloured in blue font.

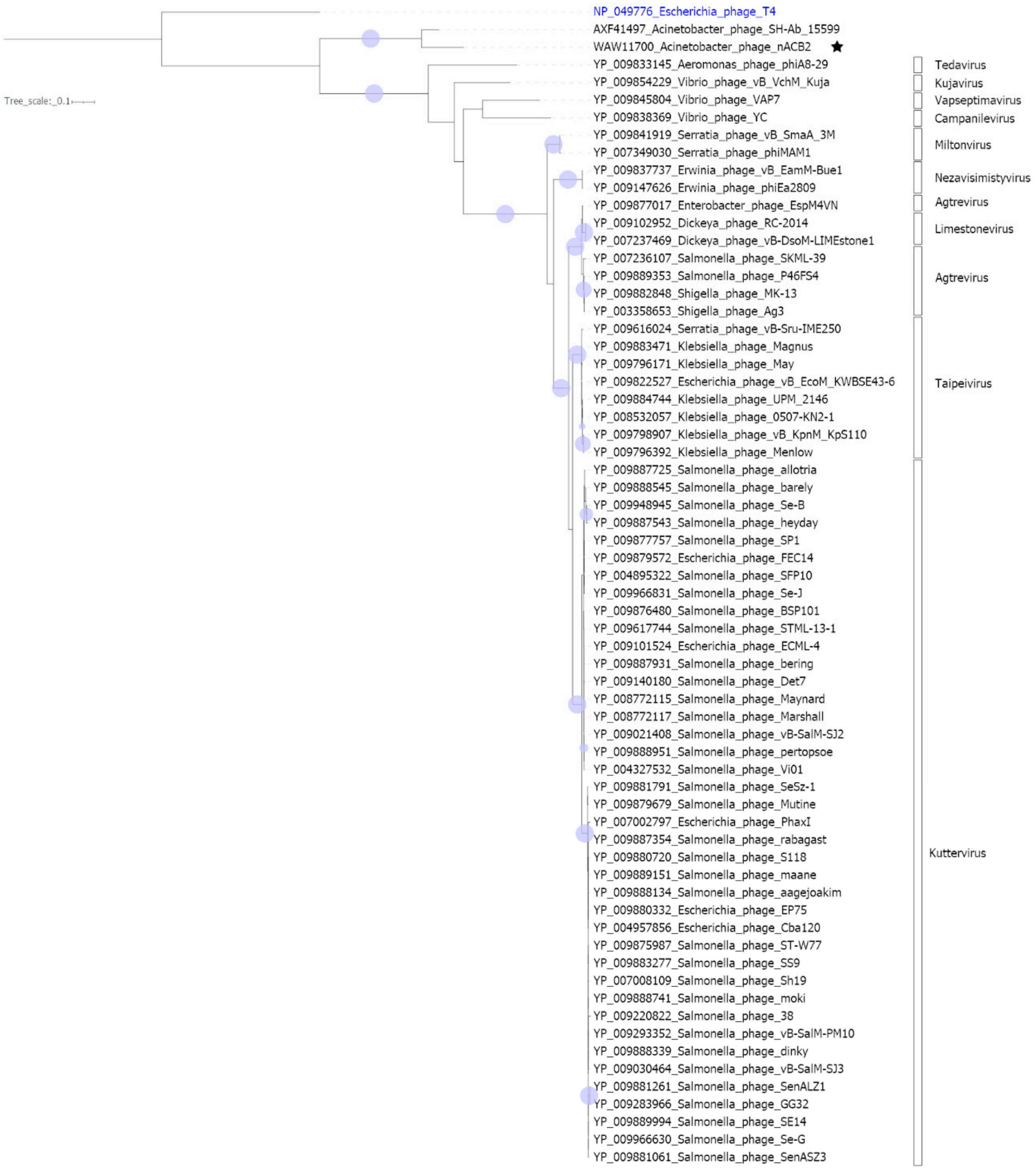

Figure S3. ***Ackermannviridae*** **phylogenetic analysis of large terminase subunit proteins.** A log-likelihood of consensus tree -13188.659 is presented with a substitution model LG+R4. Ultra-fast bootstrap >= 95% are shown as filled circles with the size proportional to the value. Bars adjacent to the labels indicate current International Committee on Taxonomy of Viruses (ICTV) genera. nACB2 is highlighted in bold font and star symbol. Outgroup sequences used to root the trees are coloured in blue font.

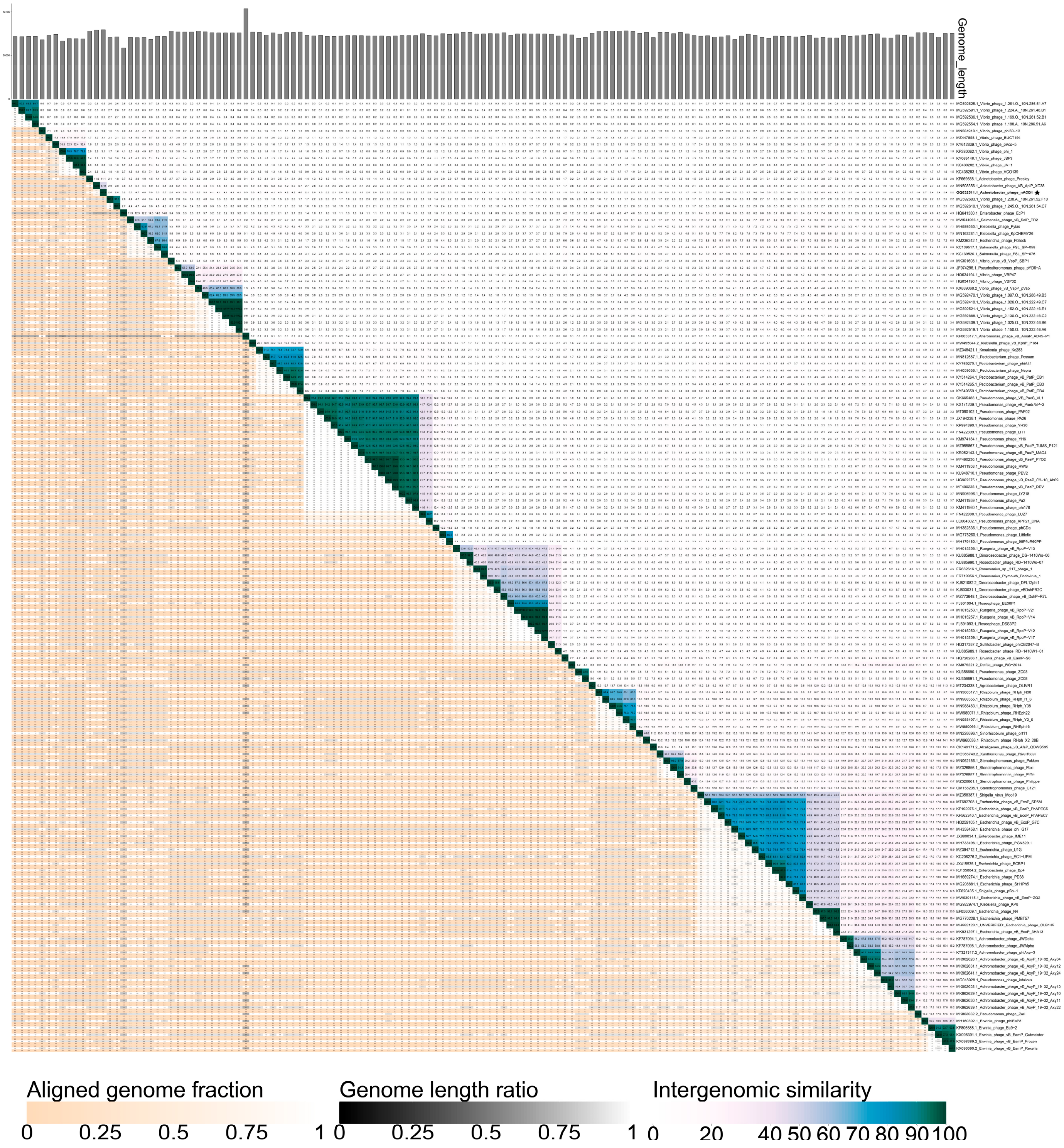

Figure S4. nACB1 VIRIDIC analysis. VIRIDIC-generated heatmap incorporating intergenomic similarity values (right half) and alignment indicators (left half and top annotation).

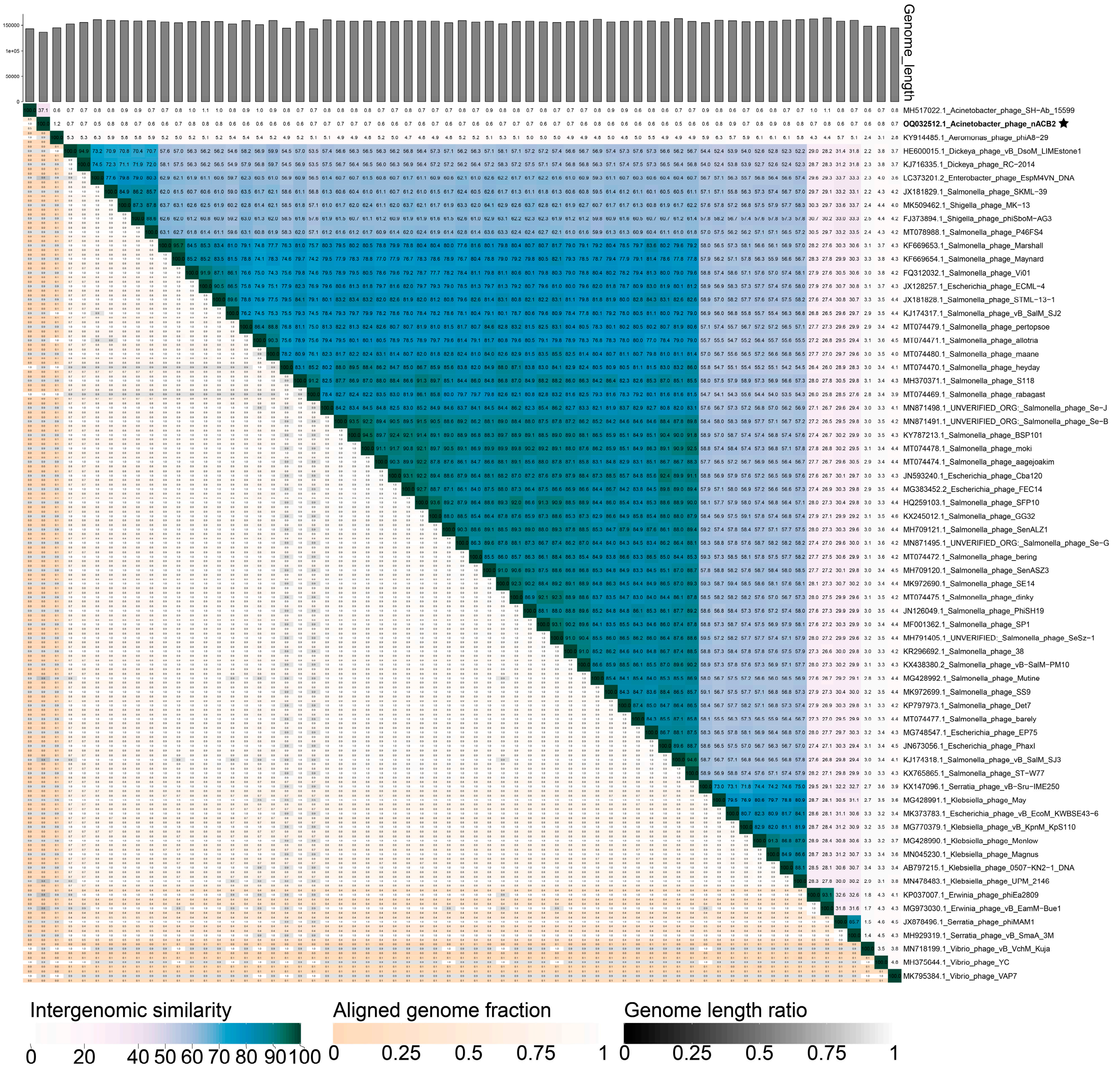

Figure S5. nACB2 VIRIDIC analysis. VIRIDIC-generated heatmap incorporating intergenomic similarity values (right half) and alignment indicators (left half and top annotation).

Table S1. **Features of nACB1 predicted CDSs.** For each CDS, the transcription start and stop position, and the coding strand is given. At protein level, the corresponding gene product size, molecular weight and pI as well as the homolog, predicted function, identity values and motifs are shown. TMD – transmembrane domain; SP – Signal peptide

ORF (+/-)	Strand	Start (bp)	Stop (bp)	Size (aa)	PI	MW (kDa)	Putative function	Best species hit (protein accession number)	E-value (% identify)	Motifs
1	reverse	566	838	91	6.43	9.631	hypothetical protein	*Acinetobacter* phage VB_ApiP_XC38 (YP_010104991.1)	3E-14 (45)	1 SP
2	forward	1,140	1,349	70	9.64	7.804	hypothetical protein	*Acinetobacter* phage Presley (YP_009007662.1)	1.7E-3 (60)	
3	forward	1,360	2,040	227	5.01	25.287	hypothetical protein	*Acinetobacter* phage VB_ApiP_XC38 (YP_010104992.1)	2E-140 (84)	
4	forward	2,033	3,670	546	5.41	61.962	terminase large subunit	*Acinetobacter* phage VB_ApiP_XC38 (YP_010104993.1)	0.0 (83)	
5	forward	3,673	4,443	257	4.64	28.557	putative tail protein	*Acinetobacter* phage VB_ApiP_XC38 (YP_010104994.1)	2E-83 (48)	
6	forward	4,592	6,796	735	4.85	82.631	portal protein	*Acinetobacter* phage VB_ApiP_XC38 (YP_010104995.1)	0.0 (80)	
7	forward	6,844	7,161	106	4.70	12.370	hypothetical protein	*Acinetobacter* phage VB_ApiP_XC38 (YP_010104996.1)	7E-23 (50)	
8	forward	7,161	8,330	390	4.21	42.625	tail measure protein	*Acinetobacter* phage VB_ApiP_XC38 (YP_010104997.1)	1E-113 (52)	
9	forward	8,343	9,503	387	5.92	42.840	hypothetical protein	*Acinetobacter* phage VB_ApiP_XC38 (YP_010104998.1)	0.0 (81)	
10	forward	9,573	10,139	189	6.54	20.526	hypothetical protein	*Acinetobacter* phage VB_ApiP_XC38 (YP_010104999.1)	1E-52 (51)	
11	forward	10,151	11,788	546	4.44	59.511	structural protein	*Acinetobacter* phage VB_ApiP_XC38 (YP_010105000.1)	0.0 (64)	
12	forward	11,844	14,237	798	4.88	89.546	hypothetical protein	*Acinetobacter* phage VB_ApiP_XC38 (YP_010105001.1)	0.0 (73)	3 TMD
13	forward	14,212	14,712	167	8.53	18.159	structural protein	*Acinetobacter* phage VB_ApiP_XC38 (YP_010105002.1)	9E-77 (70)	
14	forward	14,712	16,643	644	9.96	69.547	lytic tail fiber	*Acinetobacter* phage VB_ApiP_XC38 (YP_010105003.1)	0.0 (61)	
15	forward	16,716	26,714	3,333	5.07	364.079	virion associated RNA polymerase	*Acinetobacter* phage VB_ApiP_XC38 (YP_010105004.1)	0.0 (67)	
16	reverse	26,766	27,041	92	9.61	10.536	hypothetical protein	*Acinetobacter* phage VB_ApiP_XC38 (YP_010105005.1)	1E-33 (78)	
17	reverse	27,044	27,220	59	5.26	6.931	hypothetical protein	-		
18	reverse	27,217	27,549	111	4.76	12.806	hypothetical protein	-		
19	reverse	27,551	27,937	129	10.44	14.777	hypothetical protein	*Acinetobacter* phage VB_ApiP_XC38 (YP_010105006.1)	2E-37 (53)	
20	reverse	27,967	28,083	39	10.38	4.260	hypothetical protein	-		2 TMD
21	reverse	28,140	28,574	145	3.89	15.641	hypothetical protein	*Acinetobacter* phage VB_ApiP_XC38 (YP_010105008.1)	8E-12 (38)	
22	reverse	28,540	29,067	176	5.68	18.941	crossover junction endodeoxyribonuclease	*Acinetobacter* phage VB_ApiP_XC38 (YP_010105009.1)	2E-74 (67)	
23	reverse	29,091	29,645	185	9.87	21.012	NUMOD4 motif-containing HNH endonuclease	Sphingomonas sp. ACRSK (WP_239435786.1)	7E-30 (38)	
24	reverse	29,649	29,984	112	3.95	12.141	collagen, type VI, alpha 3	*Acinetobacter* phage VB_ApiP_XC38 (YP_010105010.1)	5E-45 (71)	
25	reverse	30,020	30,724	235	7.22	25.165	ssDNA binding protein	*Acinetobacter* phage VB_ApiP_XC38 (YP_010105012.1)	9E-81 (58)	
26	reverse	30,727	31,269	181	10.48	20.853	putative HNH endonuclease	*Acinetobacter* phage VB_ApiP_XC38 (YP_010105013.1)	6E-80 (65)	
27	reverse	31,247	31,990	248	5.87	28.039	ssDNA annealing protein	*Acinetobacter* phage VB_ApiP_XC38 (YP_010105014.1)	3E-151 (79)	
28	reverse	32,053	34,215	721	5.51	82.228	DNA primase	*Acinetobacter* phage VB_ApiP_XC38 (YP_010105015.1)	0.0 (80)	
29	reverse	34,224	35,204	327	6.67	37.083	exonuclease	*Acinetobacter* phage VB_ApiP_XC38 (YP_010105017.1)	6E-165 (67)	

30	reverse	35,206	35,343	46	3.69	5.144	hypothetical protein		
31	reverse	35,388	36,446	353	4.29	40.475	ribonucleotide reductase of class Ia (Aerobic) beta subunit	*Acinetobacter* phage VB_ApiP_XC38 (YP_010105019.1)	0.0 (82)	
32	reverse	36,436	36,681	82	7.35	9.162	hypothetical protein	*Acinetobacter* phage VB_ApiP_XC38 (YP_010105020.1)	4E-17 (45)	
33	reverse	36,686	38,677	664	6.09	74.377	ribonucleoside-diphosphate reductase	*Acinetobacter* phage VB_ApiP_XC38 (YP_010105021.1)	0.0 (84)	
34	reverse	38,721	39,365	215	6.45	24.429	thymidylate synthase	*Acinetobacter* phage VB_ApiP_XC38 (YP_010105022.1)	4E-113 (75)	
35	reverse	39,369	39,617	83	3.74	9.462	hypothetical protein	*Acinetobacter* phage VB_ApiP_XC38 (YP_010105023.1)	1E-9 (41)	
36	forward	39,712	39,987	92	4.02	10.235	tail protein	*Acinetobacter* phage VB_ApiP_XC38 (YP_010105024.1)	9E-22 (58)	
37	forward	40,007	47,476	2,490	4.83	265.987	tail protein	*Acinetobacter* phage VB_ApiP_XC38 (YP_010105024.1)	0.0 (30)	
38	forward	47,500	47,802	101	4.80	11.235	hypothetical protein	unclassified *Pseudomonas* (WP_217839566.1)	3E-8 (37)	
39	forward	47,802	48,587	262	4.89	28.269	hypothetical protein	*Burkholderia* sp. Bp9099 (WP_124920738.1)	6E-3 (26)	
40	reverse	48,622	48,816	65	4.37	7.285	hypothetical protein	*Acinetobacter* phage VB_ApiP_XC38 (YP_010105028.1)	3E-9 (50)	
41	forward	48,888	49,475	196	9.06	21.769	endolysin	*Acinetobacter* phage VB_ApiP_XC38 (YP_010105029.1)	3E-118 (84)	
42	forward	49,477	49,872	132	4.49	14.969	Rz-like spanin	*Acinetobacter* phage VB_ApiP_XC38 (YP_010105030.1)	7E-31 (44)	1 TMD
43	forward	49,872	49,991	40	6.04	4.300	hypothetical protein	-		1 TMD
44	forward	49,992	50,570	193	6.37	20.697	hypothetical protein	*Acinetobacter* phage VB_ApiP_XC38 (YP_010105032.1)	6E-68 (57)	
45	reverse	50,659	51,333	225	4.33	25.191	nucleotide diphosphate associated with X (NUDIX) protein	*Acinetobacter* phage VB_ApiP_XC38 (YP_010105033.1)	1E-60 (47)	
46	reverse	51,330	51,695	122	10.26	13.973	hypothetical protein	-		1 TMD
47	reverse	51,762	52,127	122	6.82	13.803	hypothetical protein	*Acinetobacter* phage VB_ApiP_XC38 (YP_010105035.1)	3E-9 (35)	
48	reverse	52,108	52,452	115	9.51	12.657	hypothetical protein	-		1 TMD
49	reverse	52,449	53,057	203	5.13	23.027	AAA family ATPase	*Acinetobacter* phage VB_ApiP_XC38 (YP_010105036.1)	2E-33 (35)	
50	reverse	53,054	53,353	100	4.69	10.974	hypothetical protein	-		
51	reverse	53,392	53,559	56	7.79	6.190	holin	*Acinetobacter* phage VB_ApiP_XC38 (YP_010105038.1)	1E-21 (74)	
52	forward	53,826	54,149	108	4.67	10.485	head fiber protein	*Bifidobacterium longum* (WP_118088547.1)	3E-7 (56)	
53	reverse	54,174	54,797	208	7.17	23.479	DNA polymerase	*Acinetobacter* phage VB_ApiP_XC38 (YP_010105045.1)	5E-104 (75)	
54	reverse	54,934	55,506	191	9.30	21.765	TPA: homing endonuclease	Podoviridae sp. ctXdu7 (DAE91961.1)	3E-66 (56)	
55	reverse	55,647	57,641	665	5.23	74.905	DNA polymerase	*Acinetobacter* phage VB_ApiP_XC38 (YP_010105045.1)	0.0 (74)	
56	reverse	57,638	58,159	174	5.68	20.274	hypothetical protein	*Acinetobacter* phage VB_ApiP_XC38 (YP_010105047.1)	4E-63 (55)	
57	reverse	58,159	59,382	408	5.98	44.903	DNA helicase	*Acinetobacter* phage VB_ApiP_XC38 (YP_010105048.1)	2E-124 (50)	
58	reverse	59,436	60,671	412	4.91	46.528	AAA family ATPase	*Acinetobacter* baumannii (UVM84354.1)	4E-80 (45)	
59	reverse	60,709	61,923	405	5.29	45.353	metallopeptidase domain protein	*Bacteriophage sp.* (YP_010105049.1)	6e-101 (41)	
60	reverse	61,910	62,323	138	4.83	15.784	hypothetical protein	*Acinetobacter* phage VB_ApiP_XC38 (YP_010105050.1)	3E-30 (47)	
61	reverse	62,368	63,492	375	5.85	41.127	MoxR-like ATPase protein	*Acinetobacter* phage VB_ApiP_XC38 (YP_010105051.1)	8E-138 (51)	
62	reverse	63,489	63,665	59	4.59	6.857	hypothetical protein	*Acinetobacter* phage VB_ApiP_XC38 (YP_010105052.1)	1E-16 (65)	
63	reverse	63,655	63,942	96	10.56	10.513	hypothetical protein	*Acinetobacter* phage VB_ApiP_XC38 (YP_010105053.1)	3E-30 (55)	
64	reverse	64,057	64,389	111	3.97	12.668	hypothetical protein	-		
65	reverse	64,386	64,706	107	4.03	12.783	hypothetical protein	-		
66	reverse	65,836	66,651	272	9.11	29.644	hypothetical protein	*Acinetobacter* phage VB_ApiP_XC38 (YP_010105059.1)	2E-162 (83)	

67	reverse	66,641	66,898	86	10.23	9.278	hypothetical protein			2 TMD
68	reverse	67,016	67,615	200	4.74	21.455	hypothetical protein	*Nissabacter archeti* (WP_072931907.1)	3E-7 (34)	
69	reverse	67,636	67,749	38	6.03	4.124	hypothetical protein	-		
70	reverse	67,810	68,148	113	5.14	13.388	hypothetical protein	-		
71	reverse	68,587	68,787	67	9.77	7.312	hypothetical protein	*Acinetobacter* phage VB_ApiP_XC38 (YP_010105064.1)	1E-19 (66)	
72	reverse	68,837	69,130	98	8.54	11.554	hypothetical protein	-		
73	reverse	69,123	69,404	94	8.85	10.864	hypothetical protein	-		
74	reverse	69,401	69,694	98	4.97	11.669	hypothetical protein	-		
75	reverse	69,758	70,426	223	4.43	24.358	hypothetical protein	*Acinetobacter* phage VB_ApiP_XC38 (YP_010105068.1)	2E-9 (29)	
76	reverse	70,440	70,745	102	5.02	11.453	hypothetical protein	-		
77	reverse	70,727	71,032	102	6.49	12.157	hypothetical protein	-		
78	reverse	71,014	71,190	59	3.90	6.692	hypothetical protein	-		
79	reverse	71,276	71,548	91	4.52	9.996	hypothetical protein	-		
80	reverse	71,568	71,846	93	5.82	10.834	hypothetical protein	-		
81	reverse	71,881	72,192	104	5.83	11.640	hypothetical protein	-		
82	reverse	72,167	72,433	89	5.79	10.032	hypothetical protein	-		
83	reverse	72,523	72,807	95	3.65	10.570	hypothetical protein	-		
84	reverse	72,807	73,388	194	9.95	21.697	putative HNH homing endonuclease	*Acinetobacter* phage VB_ApiP_XC38 (YP_010105074.1)	7E-104 (74)	
85	reverse	73,466	74,698	411	5.64	45.895	DNA-dependent RNA polymerase	Bacteriophage sp. (UVM84336.1)	4E-137 (49)	
86	reverse	74,716	74,985	90	4.17	10.693	hypothetical protein	-		
87	reverse	74,954	75,934	327	8.20	37.328	RNA polymerase	*Acinetobacter* phage VB_ApiP_XC38 (YP_010105079.1)	2E-163 (67)	
88	reverse	75,992	76,351	120	4.79	12.821	hypothetical protein	*Acinetobacter* phage VB_ApiP_XC38 (YP_010105080.1)	2E-15 (41)	
89	reverse	76,459	76,905	149	8.00	17.479	hypothetical protein	-		
90	reverse	76,977	77,249	91	4.09	10.479	hypothetical protein	-		
91	reverse	77,454	77,834	127	8.62	14.522	hypothetical protein	-		
92	reverse	77,837	77,998	54	8.66	5.892	hypothetical protein	-		
93	reverse	78,000	78,488	163	6.99	18.184	hypothetical protein	*Acinetobacter* phage VB_ApiP_XC38 (YP_010105085.1)	2E-18 (32)	
94	reverse	78,680	78,862	61	11.02	7.296	hypothetical protein	-		
95	reverse	78,951	79,247	99	4.35	11.188	hypothetical protein	*Acinetobacter* phage VB_ApiP_XC38 (YP_010105086.1)	3E-16 (46)	

Table S2. **Features of nACB2 predicted CDSs.** For each CDS, the transcription start and stop position, and the coding strand is given. At protein level, the corresponding gene product size, molecular weight and pI as well as the homolog, predicted function, identity values and motifs are shown. TMD – transmembrane domain; SP – Signal peptide

ORF (+/-)	**Strand**	**Start (bp)**	**Stop (bp)**	**Size (aa)**	**PI**	**MW (kDa)**	**Putative function**	**Best species hit (protein accession number)**	**E-value (% identify)**	**Motifs**
1	forward	1,115	1,810	232	5.49	25.846	hypothetical protein	*Acinetobacter* phage SH-Ab 15599 (AXF41374.1)	5E-101 (59)	-
2	forward	1,922	2,890	323	6.08	36.280	clamp loader subunit	*Acinetobacter* phage SH-Ab 15599 (AXF41375.1)	2E-167 (67)	
3	forward	2,995	3,429	145	10.12	16.861	DNA polymerase	*Synechococcus phage* S-H9-1 (YP_010669614.1)	2E-56 (60)	
4	forward	3,431	3,892	154	7.58	18.081	hypothetical protein	*Acinetobacter* phage SH-Ab 15599 (AXF41377.1)	5E-48 (56)	
5	forward	3,978	4,778	267	5.32	31.310	hypothetical protein	*Acinetobacter* phage SH-Ab 15599 (AXF41378.1)	4E-87 (48)	
6	forward	4,940	5,635	232	4.64	26.016	hypothetical protein	*Acinetobacter* phage SH-Ab 15599 (AXF41379.1)	2E-85 (61)	
7	forward	5,696	6,043	116	10.11	13.290	hypothetical protein	*Acinetobacter* phage SH-Ab 15599 (AXF41380.1)	3E-6 (44)	1 TMD
8	forward	6,151	7,203	351	4.97	39.861	hypothetical protein	*Acinetobacter* phage SH-Ab 15599 (AXF41382.1)	0.0 (72)	
9	forward	7,268	7,966	233	8.45	25.708	hypothetical protein	*Acinetobacter* phage SH-Ab 15599 (AXF41383.1)	4E-33 (34)	
10	forward	7,991	8,956	322	6.62	38.016	hypothetical protein	*Acinetobacter* phage SH-Ab 15599 (AXF41385.1)	4E-151 (67)	
11	forward	9,037	10,149	371	5.76	42.640	thymidylate synthase	*Delftia* phage PhiW-*14* (YP_003359084.1)	5E-78 (40)	
12	forward	10,149	11,321	391	4.55	45.496	hypothetical protein	*Acinetobacter* phage SH-Ab 15599 (AXF41387.1)	1E-126 (50)	
13	forward	11,324	12,403	360	5.39	40.595	RecA-like recombination protein	*Acinetobacter* phage SH-Ab 15599 (AXF41388.1)	0.0 (69)	
14	forward	12,453	13,868	472	5.22	53.344	DNA primase/helicase	*Acinetobacter* phage SH-Ab 15599 (AXF41389.1)	0.0 (75)	
15	forward	13,951	17,232	1094	9.04	121.071	hypothetical protein	*Acinetobacter* phage SH-Ab 15599 (AXF41390.1)	4E-65 (62)	
16	forward	17,331	18,713	461	5.08	52.009	putative DNA ligase	*Acinetobacter* phage SH-Ab 15599 (AXF41391.1)	0.0 (66)	
17	forward	18,892	19,146	85	8.36	9.119	hypothetical protein	*Acinetobacter* phage SH-Ab 15599 (AXF41489.1)	1E-11 (44)	
18	forward	19,282	19,587	102	9.56	11.388	hypothetical protein	-		
19	forward	19,681	21,438	586	5.34	64.826	baseplate wedge subunit	*Acinetobacter* phage SH-Ab 15599 (AXF41393.1)	0.0 (67)	
20	forward	21,435	23,234	600	5.36	68.104	hypothetical protein	*Acinetobacter* phage SH-Ab 15599 (AXF41394.1)	0.0 (57)	
21	forward	23,260	23,772	171	7.15	18.729	tail sheath	*Ralstonia* phage RSP15 (YP_009277000.1)	1E-12 (42)	
22	forward	23,820	24,116	99	6.36	10.856	tail sheath	*Ralstonia* phage RSP15 (YP_009277000.1)	2E-15 (42)	
23	forward	24,123	25,730	536	4.44	59.701	tail sheath protein	*Acinetobacter* phage SH-Ab 15599 (AXF41397.1)	0.0 (77)	
24	forward	25,776	26,303	176	4.51	19.825	tail tube	*Delftia phage* PhiW-14 YP_003358899.1)	5E-49 (48)	
25	forward	26,316	27,863	516	5.84	59.511	portal protein	*Acinetobacter* phage SH-Ab 15599 (AXF41399.1)	0.0 (80)	
26	forward	27,853	28,053	67	4.69	7.012	hypothetical protein	*Acinetobacter* phage SH-Ab 15599 (AXF41400.1)	1E-7 (52)	
27	forward	28,064	28,345	94	11.06	10.601	prohead core protein	*Klebsiella* phage 0507-KN2-1 (YP_008532063.1)	1E-44 (43)	
28	forward	28,342	29,010	223	4.89	25.152	prohead protease	*Acinetobacter* phage SH-Ab 15599 (AXF41402.1)	2E-129 (78)	
29	forward	29,069	29,821	251	3.87	26.717	scaffold prohead core protein	*Acinetobacter* phage SH-Ab 15599 (AXF41403.1)	1E-84 (57)	
30	forward	29,887	31,209	441	4.77	47.253	major capsid protein	*Acinetobacter* phage SH-Ab 15599 (AXF41404.1)	0.0 (84)	

31	reverse	31,270	32,310	347	8.46	40.660	hypothetical protein	*Acinetobacter* phage SH-Ab 15599 (AXF41405.1)	6E-33 (32)	
32	reverse	32,422	32,808	129	4.05	14.043	hypothetical protein	-		
33	reverse	32,903	33,214	104	5.87	12.108	hypothetical protein	-		
34	reverse	33,211	33,495	95	7.53	11.177	hypothetical protein	-		
35	reverse	33,495	33,968	158	4.32	17.963	hypothetical protein	-		
36	reverse	33,981	34,322	114	5.12	13.533	hypothetical protein	-		
37	reverse	34,356	34,862	169	4.36	19.430	hypothetical protein	*Acinetobacter* phage SH-Ab 15599 (AXF41416.1)	3E-24 (44)	
38	reverse	34,862	35,692	277	4.75	32.554	hypothetical protein	-		2 TMD
39	reverse	35,689	36,108	104	4.91	16.426	hypothetical protein	-		
40	forward	36,269	36,646	126	4.67	14.103	hypothetical protein	-		
41	reverse	36,693	37,700	336	6.30	38.959	hypothetical protein	*Acinetobacter* phage SH-Ab 15599 (AXF41420.1)	4E-33 (29)	
42	forward	37,867	38,142	92	9.82	9.637	DNA-binding protein HU-beta	*Acinetobacter* phage SH-Ab 15599 (AXF41421.1)	6E-31 (62)	
43	reverse	38,182	38,346	55	10.66	6.204	hypothetical protein	*Actinomycetia bacterium* (MCH9735535.1)	4E-13 (62)	
44	reverse	38,392	38,637	82	7.01	9.481	hypothetical protein	-		1 TMD
45	reverse	38,637	38,969	111	6.26	12.578	DUF3307 domain-containing protein	*Acinetobacter baumannii* (MCJ9373407.1)	3E-17 (41)	3 TMD
46	reverse	38,966	39,493	176	5.66	19.737	hypothetical protein	*Acinetobacter* phage SH-Ab 15599 (AXF41424.1)	5E-38 (48)	
47	reverse	39,509	40,447	313	9.37	35.237	hypothetical protein	*Acinetobacter* phage SH-Ab 15599 (AXF41425.1)	1E-56 (35)	
48	reverse	40,554	41,123	190	5.19	21.577	hypothetical protein	*Acinetobacter* phage SH-Ab 15599 (AXF41427.1)	2E-80 (62)	2 TMD
49	reverse	41,136	42,647	504	6.18	58.421	DNA polymerase	*Acinetobacter* phage SH-Ab 15599 (AXF41428.1)	0.0 (78)	
50	reverse	42,773	43,684	304	8.96	34.379	hypothetical protein	*Acinetobacter* phage SH-Ab 15599 (AXF41429.1)	6E-163 (74)	
51	reverse	43,782	45,053	424	4.90	48.816	DNA polymerase	*Acinetobacter* phage SH-Ab 15599 (AXF41430.1)	0.0 (71)	
52	forward	45,133	45,642	170	3.99	19.104	tail completion and sheath stabilizer protein	*Acinetobacter* phage SH-Ab 15599 (AXF41431.1)	4E-73 (60)	
53	reverse	45,672	45,851	60	4.05	6.722	hypothetical protein	*Acinetobacter puyangensis* (WP_097078444.1)	3E-9 (47)	
54	reverse	45,940	46,719	260	6.53	30.275	putative exonuclease	*Acinetobacter* phage SH-Ab 15599 (AXF41432.1)	5E-139 (70)	
55	reverse	46,721	47,062	114	7.78	13.094	hypothetical protein	-		
56	reverse	47,059	47,220	54	11.10	5.667	hypothetical protein	-		2 TMD
57	reverse	47,220	47,693	158	7.29	18.517	putative repair and recombination protein	*Acinetobacter* phage SH-Ab 15599 (AXF41435.1)	2E-55 (54)	
58	reverse	47,872	48,576	235	5.21	26.414	hypothetical protein	*Acinetobacter* phage SH-Ab 15599 (AXF41436.1)	9E-61 (44)	
59	reverse	48,578	49,318	247	5.59	27.689	hypothetical protein	*Acinetobacter* phage SH-Ab 15599 (AXF41437.1)	7E-101 (58)	
60	reverse	49,311	49,646	112	5.02	12.418	hypothetical protein	*Acinetobacter* phage SH-Ab 15599 (AXF41438.1)	4E-43 (59)	3 TMD
61	reverse	49,760	51,100	447	6.38	51.352	topoisomerase II medium subunit	*Acinetobacter* phage SH-Ab 15599 (AXF41439.1)	7E-172 (57)	
62	reverse	51,103	51,507	135	8.78	15.519	hypothetical protein	-		
63	reverse	51,507	51,857	117	4.28	13.509	hypothetical protein	-		
64	reverse	51,858	52,163	102	4.98	11.548	hypothetical protein	-		
65	reverse	52,160	52,498	113	4.76	12.833	hypothetical protein	-		
66	reverse	52,495	52,818	108	5.92	12.472	hypothetical protein	*Acinetobacter* phage SH-Ab 15599 (AXF41441.1)	2E-12 (45)	
67	reverse	52,844	53,272	143	4.37	16.543	hypothetical protein	*Acinetobacter* phage SH-Ab 15599 (AXF41442.1)	3E-21 (36)	

68	reverse	53,275	54,075	267	5.15	30.234	hypothetical protein	*Acinetobacter* phage SH-Ab 15599 (AXF41443.1)	2E-21 (35)	
69	reverse	54,188	54,400	71	10.58	8.345	hypothetical protein	*Acinetobacter* phage SH-Ab 15599 (AXF41444.1)	2E-19 (63)	
70	reverse	54,412	54,948	179	5.87	19.937	DNA polymerase III subunit epsilon	*Oxalobacter* sp. (MBR6000052.1)	1E-35 (47)	
71	reverse	54,998	55,177	60	8.76	7.123	hypothetical protein	-		
72	reverse	55,174	57,021	616	5.85	70.037	DNA topoisomerase	*Acinetobacter* phage SH-Ab 15599 (AXF41446.1)	0.0 (69)	
73	reverse	57,014	57,175	54	4.17	6.354	hypothetical protein	-		
74	reverse	57,176	57,823	216	4.82	23.947	hypothetical protein	*Candidatus Methanofastidiosa* archaeon (NMC57943.1)	1E-21 (43)	
75	reverse	57,893	59,377	495	8.08	57.353	hypothetical protein	*Acinetobacter* phage SH-Ab 15599 (AXF41449.1)	0.0 (62)	
76	reverse	59,414	59,719	102	9.72	11.608	DUF4326 domain-containing protein	Acidobacteria bacterium (MBS1789655.1)	2E-20 (38)	
77	reverse	59,716	60,027	104	11.15	11.412	hypothetical protein	*Acinetobacter* phage SH-Ab 15599 (AXF41453.1)	9E-8 (51)	1 SP
78	reverse	60,017	60,355	113	10.05	13.055	hypothetical protein	*Acinetobacter* phage SH-Ab 15599 (AXF41454.1)	3E-22 (50)	
79	reverse	60,393	60,533	47	4.78	5.610	hypothetical protein	-		
80	forward	61,760	62,032	91	5.45	10.634	hypothetical protein	-		1 TMD
81	forward	62,076	62,486	137	5.07	15.943	hypothetical protein	-		1 TMD
82	forward	62,476	63,036	187	6.75	21.228	putative RNaseH ribonuclease	*Acinetobacter* phage SH-Ab 15599 (AXF41458.1)	1E-87 (67)	
83	forward	63,033	63,623	197	5.43	22.741	hypothetical protein	*Acinetobacter* phage SH-Ab 15599 (AXF41459.1)	7E-76 (59)	
84	forward	63,627	63,953	109	8.04	12.419	hypothetical protein	-		
85	forward	63,947	64,090	48	9.94	5.814	hypothetical protein	-		
86	reverse	64,124	65,272	383	4.35	43.480	hypothetical protein	*Acinetobacter* phage SH-Ab 15599 (AXF41460.1)	7E-48 (46)	
87	forward	65,360	65,764	135	4.86	14.733	hypothetical protein	*Acinetobacter* phage SH-Ab 15599 (AXF41461.1)	6E-17 (34)	1 SP
88	forward	65,768	66,880	371	5.15	42.231	endonuclease	*Acinetobacter* phage SH-Ab 15599 (AXF41462.1)	0.0 (69)	
89	forward	66,885	67,235	117	7.65	13.641	hypothetical protein	*Acinetobacter* phage SH-Ab 15599 (AXF41463.1)	3E-17 (39)	
90	forward	67,235	69,373	713	4.90	81.855	recombination-related endonuclease	*Acinetobacter* phage SH-Ab 15599 (AXF41464.1)	0.0 (51)	
91	forward	69,366	69,581	72	4.56	8.182	hypothetical protein	-		
92	forward	69,645	69,839	65	7.03	7.345	hypothetical protein	-		
93	forward	69,836	70,162	109	7.79	12.333	hypothetical protein	-		
94	forward	70,268	71,293	342	8.82	39.962	DNA primase subunit	*Acinetobacter* phage SH-Ab 15599 (AXF41467.1)	1E-156 (65)	
95	forward	71,304	71,633	110	4.75	12.811	hypothetical protein	-		
96	forward	71,721	72,116	132	5.35	14.441	hypothetical protein	*Acinetobacter* phage SH-Ab 15599 (AXF41469.1)	2E-3 (23)	
97	forward	72,189	74,750	854	5.63	97.901	hypothetical protein	*Acinetobacter* phage SH-Ab 15599 (AXF41471.1)	0.0 (40)	
98	forward	74,811	75,935	375	9.69	41.742	RIIB protein	*Acinetobacter* phage SH-Ab 15599 (AXF41472.1)	6E-130 (51)	
99	forward	75,976	76,353	126	7.81	14.215	hypothetical protein	*Acinetobacter* phage SH-Ab 15599 (AXF41473.1)	2E-26 (45)	
100	forward	76,380	76,772	131	4.20	14.870	hypothetical protein	*Acinetobacter* phage SH-Ab 15599 (AXF41474.1)	3E-18 (32)	
101	forward	76,763	77,575	271	9.54	29.956	protease	*Acinetobacter* phage SH-Ab 15599 (AXF41475.1)	1E-102 (57)	1 TMD
102	forward	77,578	77,994	139	4.68	16.027	hypothetical protein	*Delftia* sp. BR1 (WP_151020094.1)	3E-33 (47)	1 SP
103	forward	78,077	78,229	51	7.79	5.388	hypothetical protein			2 TMD
104	forward	78,229	78,690	154	5.43	17.676	hypothetical protein			

105	reverse	78,647	78,910	88	4.54	9.660	hypothetical protein	*Acinetobacter* phage SH-Ab 15599 (AXF41478.1)	3E-14 (43)	
106	forward	79,024	79,578	185	6.51	21.172	hypothetical protein DP-phiW-14_gp129	*Delftia* phage PhiW-14 (YP_003358982.1)	1E-23 (37)	
107	forward	79,578	79,865	96	5.66	10.624	hypothetical protein	-		
108	forward	79,858	80,175	106	4.67	11.208	hypothetical protein	-		
109	forward	80,168	80,311	48	10.37	5.269	hypothetical protein	-		
110	forward	81,016	81,315	100	10.03	12.121	hypothetical protein	*Acinetobacter* phage SH-Ab 15599 (AXF41486.1)	5E-14 (38)	
111	forward	81,305	81,511	69	10.88	8.103	hypothetical protein	-		
112	forward	81,610	82,212	201	5.35	22.031	hypothetical protein	*Acinetobacter* phage SH-Ab 15599 (AXF41489.1)	7E-54 (49)	
113	forward	82,339	82,668	110	3.65	12.024	hypothetical protein	*Acinetobacter* phage SH-Ab 15599 (AXF41483.1)	4E-5 (26)	
114	forward	82,861	83,187	109	5.37	12.442	hypothetical protein	-		
115	forward	83,180	83,491	104	8.54	12.245	hypothetical protein	-		
116	forward	83,484	83,843	120	8.86	13.599	hypothetical protein	-		
117	forward	83,840	84,046	69	9.71	7.968	hypothetical protein	-		
118	reverse	84,142	85,926	595	4.93	66.629	terminase DNA packaging enzyme large subunit	*Acinetobacter* phage SH-Ab 15599 (AXF41497.1)	0.0 (73)	
119	reverse	85,916	86,428	171	4.22	18.668	hypothetical protein	*Acinetobacter* phage SH-Ab 15599 (AXF41498.1)	1E-70 (70)	
120	reverse	86,421	87,179	253	5.07	28.369	tail connector protein	*Acinetobacter* phage SH-Ab 15599 (AXF41499.1)	1E-111 (63)	
121	reverse	87,181	87,807	209	3.91	23.560	neck protein	*Acinetobacter* phage SH-Ab 15599 (AXF41500.1)	7E-93 (62)	
122	reverse	87,829	88,611	261	4.60	29.564	hypothetical protein	*Acinetobacter* phage SH-Ab 15599 (AXF41501.1)	9E-170 (84)	
123	reverse	88,598	88,798	67	8.82	7.696	hypothetical protein	*Acinetobacter* phage SH-Ab 15599 (AXF41502.1)	6E-10 (41)	1 TMD
124	reverse	88,808	93,613	1602	4.57	174.722	hypothetical protein	*Acinetobacter* phage SH-Ab 15599 (AXF41503.1)	0.0 (68)	
125	reverse	93,696	96,512	939	4.45	99.508	hypothetical protein	*Acinetobacter* phage SH-Ab 15599 (AXF41504.1)	0.0 (61)	
126	reverse	96,535	96,942	136	10.16	14.772	hypothetical protein	*Acinetobacter* phage SH-Ab 15599 (AXF41505.1)	1E-29 (42)	
127	reverse	96,942	99,407	822	4.24	87.328	hypothetical protein	*Acinetobacter* phage SH-Ab 15599 (AXF41506.1)	0.0 (42)	
128	forward	99,479	99,844	122	5.40	13.877	hypothetical protein	*Acinetobacter* phage SH-Ab 15599 (AXF41507.1)	1E-26 (40)	
129	reverse	99,841	100,212	124	5.93	14.227	endolysin	*Acinetobacter* phage SH-Ab 15599 (AXF41508.1)	1E-54 (68)	
130	reverse	100,205	101,647	481	5.20	52.189	tail associated lysozyme	*Acinetobacter* phage SH-Ab 15599 (AXF41509.1)	0.0 (68)	
131	reverse	101,647	102,600	318	7.73	34.153	hypothetical protein	*Acinetobacter* phage SH-Ab 15599 (AXF41510.1)	0.0 (83)	
132	reverse	102,678	103,016	113	4.51	12.594	hypothetical protein	*Acinetobacter* phage SH-Ab 15599 (AXF41512.1)	3E-46 (64)	
133	reverse	103,205	103,846	214	4.45	23.433	hypothetical protein	*Acinetobacter* phage SH-Ab 15599 (AXF41514.1)	3E-37 (46)	
134	reverse	103,848	104,270	141	6.34	15.877	cytidine deaminase	*Acinetobacter* phage SH-Ab 15599 (AXF41515.1)	2E-31 (55)	
135	reverse	104,267	104,476	70	8.04	8.022	hypothetical protein	*Acinetobacter* phage SH-Ab 15599 (AXF41516.1)	0,000004 (44)	
136	reverse	104,478	105,287	270	6.22	31.073	hypothetical protein	*Acinetobacter* phage SH-Ab 15599 (AXF41517.1)	5E-105 (54)	
137	reverse	105,330	105,569	80	8.96	8.911	hypothetical protein	*Acinetobacter* phage SH-Ab 15599 (AXF41519.1)	2E-12 (49)	
138	reverse	105,566	105,859	98	6.09	11.025	hypothetical protein	*Acinetobacter* proteolyticus (WP_004653838.1)	1E-10 (39)	
139	reverse	105,859	107,568	570	5.83	64.468	ATP-dependent helicase	*Acinetobacter* phage SH-Ab 15599 (AXF41520.1)	0.0 (68)	
140	reverse	107,572	108,234	221	5.56	25.473	exonuclease A	*Acinetobacter* phage SH-Ab 15599 (AXF41521.1)	3E-90 (61)	
141	reverse	108,312	109,145	278	4.56	30.859	hypothetical protein	*Acinetobacter* phage SH-Ab 15599 (AXF41522.1)	1E-73 (46)	

142	reverse	109,217	111,205	663	7.44	72.304	hypothetical protein	*Acinetobacter* phage SH-Ab 15599 (AXF41523.1)	0.0 (52)	
143	reverse	111,202	112,509	436	4.63	50.096	hypothetical protein	*Acinetobacter* phage SH-Ab 15599 (AXF41524.1)	0.0 (73)	3 TMD
144	reverse	112,506	113,039	178	5.14	20.828	baseplate wedge component	*Acinetobacter* phage SH-Ab 15599 (AXF41525.1)	9E-80 (61)	
145	reverse	113,176	114,087	304	5.61	33.226	putative tail tube associated base plate protein	*Acinetobacter* phage SH-Ab 15599 (AXF41526.1)	4E-172 (76)	
146	reverse	114,125	114,673	183	10.12	21.574	head completion protein	*Acinetobacter* phage SH-Ab 15599 (AXF41527.1)	7E-74 (68)	
147	reverse	114,673	114,993	107	5.38	12.153	DUF6527 family protein	*Acinetobacter* tandoii (WP_016167361.1)	4E-61 (84)	
148	reverse	114,990	115,568	193	5.45	22.020	tail tube monomer	*Acinetobacter* phage SH-Ab 15599 (AXF41529.1)	5E-94 (63)	
149	forward	115,626	116,297	224	10.30	25.928	DNA end protector protein	*Acinetobacter* phage SH-Ab 15599 (AXF41530.1)	1E-111 (69)	
150	reverse	116,275	116,901	209	5.17	24.229	hypothetical protein	*Acinetobacter* phage SH-Ab 15599 (AXF41531.1)	9E-68 (56)	
151	reverse	116,971	118,029	353	4.79	39.300	single stranded DNA-binding protein	*Acinetobacter* phage SH-Ab 15599 (AXF41532.1)	7E-148 (60)	
152	reverse	118,173	118,427	85	4.09	9.424	hypothetical protein	*Acinetobacter* phage SH-Ab 15599 (AXF41533.1)	2E-33 (71)	
153	reverse	118,424	118,672	83	6.86	9.175	hypothetical protein	*Acinetobacter* phage SH-Ab 15599 (AXF41534.1)	7E-25 (55)	
154	reverse	118,673	119,110	146	4.89	16.849	hypothetical protein	*Acinetobacter* phage SH-Ab 15599 (AXF41535.1)	2E-31 (44)	
155	reverse	119,136	119,693	186	5.95	20.548	hypothetical protein	*Acinetobacter* phage SH-Ab 15599 (AXF41536.1)	2E-47 (56)	
156	reverse	119,697	119,84	48	3.86	5.526	hypothetical protein	*Acinetobacter* phage SH-Ab 15599 (AXF41537.1)	0.002 (43)	
157	reverse	119,943	120,659	239	4.10	25.859	hypothetical protein	*Acinetobacter* phage SH-Ab 15599 (AXF41538.1)	2E-71 (60)	
158	reverse	120,75	121,793	348	4.91	40.685	ribonucleotide reductase	*Acinetobacter* phage SH-Ab 15599 (AXF41539.1)	0.0 (77)	
159	reverse	121,800	124,418	873	5.93	98.251	ribonucleotide-diphosphate reductase subunit alpha	*Acinetobacter* phage SH-Ab 15599 (AXF41540.1)	0.0 (80)	
160	forward	124,551	125,324	258	4.60	29.625	baseplate hub subunit	*Acinetobacter* phage SH-Ab 15599 (AXF41541.1)	6E-89 (53)	
161	reverse	125,503	125,835	111	8.71	12.778	hypothetical protein	-		
162	reverse	125,905	126,438	178	4.79	20.278	hypothetical protein	-		
163	reverse	126,476	127,081	202	9.03	22.578	metallopeptidase	*Acinetobacter* phage SH-Ab 15599 (AXF41545.1)	3E-86 (65)	1 TMD
164	reverse	127,227	130,271	1015	4.74	110.401	hypothetical protein	*Acinetobacter calcoaceticus* (WP_227546728.1)	0.0 (47)	
165	reverse	130,492	133,131	880	5.03	94.664	hypothetical protein	*Aquamicrobium lusatiense* (WP_183831682.1)	4E-83 (33)	
166	reverse	133,197	136,220	1008	5.42	108.691	SGNH/GDSL hydrolase family protein	*Acinetobacter bereziniae* (WP_262540162.1)	8E-58 (50)	
